# Long-term suicide risk of children and adolescents with attention deficit and hyperactivity disorder – a systematic review

**DOI:** 10.1192/j.eurpsy.2022.826

**Published:** 2022-09-01

**Authors:** P. Garas, J. Balazs

**Affiliations:** 1 Semmelweis University, Mental Health Sciences School Of Ph.d. Studies, Budapest, Hungary; 2 Eotvos Lorant University, Developmental And Clinical Child Psychology, Budapest, Hungary; 3 Bjørknes University College, -, Oslo, Norway

**Keywords:** ADHD, suicidality, review, children

## Abstract

**Introduction:**

Several studies showed the high suicide risk of patients with attention deficit and hyperactivity disorder (ADHD), however most of these studies had cross-sectional design.

**Objectives:**

The aim of the current study was to review systematically those studies which investigated the suicide risk among ADHD patients with longitudinal design.

**Methods:**

The systematic search was made on OVID Medline, PsychInfo, PubMed, Scopus, and Web of Science. The search terms were (ADHD OR attention deficit hyperactivity disorder) AND (suicide OR suicidal OR suicidality) AND (follow-up OR longitudinal study OR prospective study). Inclusion criteria were: written in English; participants under 18 years at the baseline; longitudinal, prospective studies; ADHD population at the baseline and at the follow-up; suicide behavior as primary outcome. Exclusion criteria were: the study did not contain empirical data, and reviews/meta-analyses and studies which aimed to investigate the drug treatment efficacy of ADHD.

**Results:**

18 papers were included in the systematic review. 10 articles were published in the last 5 years. 9 studies enrolled children aged under 12 at baseline. The follow-up periods varied between 2 and 17 years. 17 studies found a significant positive association between ADHD diagnosis at baseline and the future suicidal behavior and/or attempts at the follow-up. The affective comorbidity showed an association with the future suicide risk.

**Conclusions:**

These results highlight the importance of screening suicidality in patient with ADHD and consider it during treatment. Further studies are needed to clarify the role of the treatment and comorbidities of ADHD in the increased suicide risk.

**Disclosure:**

No significant relationships.

